# Development and Evaluation of a Newcastle Disease Virus-like Particle Vaccine Expressing SARS-CoV-2 Spike Protein with Protease-Resistant and Stability-Enhanced Modifications

**DOI:** 10.3390/v16121932

**Published:** 2024-12-18

**Authors:** Yu Chen, Fan Tian, Shunlin Hu, Xiufan Liu

**Affiliations:** 1 Animal Infectious Disease Laboratory, College of Veterinary Medicine, Yangzhou University, Yangzhou 225012, China; 2 Jiangsu Co-Innovation Center for Prevention and Control of Important Animal Infectious Diseases and Zoonosis, Yangzhou University, Yangzhou 225012, China; 3 Jiangsu Key Laboratory of Zoonosis, Yangzhou University, Yangzhou 225012, China

**Keywords:** SARS-CoV-2, NDV, VLP, vaccine, immune response

## Abstract

The ongoing global health crisis caused by severe acute respiratory syndrome coronavirus 2 (SARS-CoV-2) necessitates the continuous development of innovative vaccine strategies, especially in light of emerging viral variants that could undermine the effectiveness of existing vaccines. In this study, we developed a recombinant virus-like particle (VLP) vaccine based on the Newcastle Disease Virus (NDV) platform, displaying a stabilized prefusion form of the SARS-CoV-2 spike (S) protein. This engineered S protein includes two proline substitutions (K986P, V987P) and a mutation at the cleavage site (RRAR to QQAQ), aimed at enhancing both its stability and immunogenicity. Using a prime-boost regimen, we administered NDV-VLP-S-3Q2P intramuscularly at different doses (2, 10, and 20 µg) to BALB/c mice. Robust humoral responses were observed, with high titers of S-protein-specific IgG and neutralizing antibodies against SARS-CoV-2 pseudovirus, reaching titers of 1:2200–1:2560 post-boost. The vaccine also induced balanced Th1/Th2 immune responses, evidenced by significant upregulation of cytokines (IFN-γ, IL-2, and IL-4) and S-protein-specific IgG1 and IgG2a. Furthermore, strong activation of CD4+ and CD8+ T cells in the spleen and lungs confirmed the vaccine’s ability to promote cellular immunity. These findings demonstrate that NDV-S3Q2P-VLP is a potent immunogen capable of eliciting robust humoral and cellular immune responses, highlighting its potential as a promising candidate for further clinical development in combating COVID-19.

## 1. Introduction

The Coronavirus disease 2019 (COVID-19) pandemic, caused by the severe acute respiratory syndrome coronavirus 2 (SARS-CoV-2), has emerged as one of the most profound global public health challenges of modern times. To date, SARS-CoV-2 has infected more than 700 million people worldwide, resulting in over 7 million deaths and triggering widespread socio-economic disruption [1,2]. The virus is primarily transmitted through respiratory droplets, and its high transmissibility, coupled with the emergence of new variants, has complicated containment efforts [3,4]. Notably, variants of concern, such as Delta and Omicron, have exhibited enhanced infectivity and potential to evade immunity, further complicating global efforts to control the pandemic [5,6,7].

Vaccination remains the most effective strategy to mitigate the pandemic, providing significant relief to healthcare systems and preventing severe disease outcomes. Several COVID-19 vaccines, developed using platforms such as mRNA, viral vectors, protein subunits, and inactivated viruses, have shown remarkable efficacy in both clinical trials and real-world applications. For example, mRNA vaccines, such as Pfizer-BioNTech’s BNT162b2 [8,9] and Moderna’s mRNA-1273 [10,11], have demonstrated over 90% efficacy in preventing symptomatic COVID-19. Similarly, viral vector vaccines like AstraZeneca’s ChAdOx1-S [12,13] and Johnson & Johnson’s Ad26.COV2.S [14,15] provide substantial protection against severe disease. Inactivated vaccines, such as Sinovac’s CoronaVac [16,17] and Sinopharm’s BBIBP-CorV [18,19], have been particularly important in regions with limited access to advanced technologies, offering a more traditional approach to immunization. Despite these successes, challenges persist, including unequal vaccine distribution, the durability of immune protection, and reduced efficacy against emerging variants.

To address these ongoing challenges, the development of next-generation vaccines is of paramount global health importance. Among the promising candidates are virus-like particles (VLPs), which have garnered significant attention as a promising platform due to their structural resemblance to native viruses and the absence of viral genetic material. This unique feature enables VLPs to elicit robust immune responses while offering a safer alternative to whole pathogen-based vaccines, such as live-attenuated viral vaccines [20,21]. Moreover, VLPs can be produced in various host systems, including prokaryotic cells, yeast, insect cells, plants, and mammalian cells, providing flexibility in production methods [22]. These diverse expression systems enable the large-scale, high-efficiency production of VLPs, making this platform highly scalable and well-suited for rapid responses during pandemics. To date, VLP vaccines have been successfully developed and approved for clinical use against several infectious diseases. For example, the hepatitis B vaccine, the first VLP-based vaccine to be approved by the US FDA in 1986, has been widely used for decades [20,23]. Human papillomavirus (HPV) vaccines, such as Gardasil and Cervarix, have proven highly effective in preventing HPV infection and related cancers [24,25]. Additionally, a VLP vaccine against hepatitis E virus (HEV), known as Hecolin, has been licensed in China, further highlighting the versatility of the VLP platform [26].

Despite the success of VLP vaccines in preventing other infectious diseases, no VLP vaccine has yet been approved for clinical use against COVID-19. However, ongoing research has shown promising results. For instance, Tan et al. developed a COVID-19 nanoparticle vaccine using the receptor-binding domain (RBD) of the spike (S) protein displayed on a synthetic VLP platform, which elicited high levels of neutralizing antibodies in mice and pigs [27]. In addition to using the RBD, S protein has also been utilized as an antigen in VLP vaccines [28]. A major challenge in using the SARS-CoV-2 S protein as an antigen lies in maintaining its structural stability, as the protein undergoes conformational changes during viral entry that can reduce its immunogenicity.

Newcastle disease virus (NDV), a negative-sense, single-stranded RNA virus from the Paramyxoviridae family, has been widely used as a viral vector in vaccine development due to its ability to induce strong immune responses and its favorable safety profile in humans [29,30]. The structural proteins of NDV—fusion (F), nucleoprotein (NP), and matrix (M)—assemble to form stable, membrane-enveloped VLPs [31,32]. In addition, the transmembrane (TM) and cytoplasmic tail (CT) domains of the NDV F protein offer ideal sites for the incorporation of foreign sequences, facilitating the fusion of heterologous proteins or antigens from other pathogens [33]. When foreign proteins are fused with the TM and CT regions of the F protein, they are incorporated into the surface of the NDV VLPs, enabling the display of critical epitopes. This feature makes NDV VLPs a highly versatile platform for the development of vaccines against a wide range of infectious diseases.

A common stabilization strategy involves introducing two proline substitutions (K986P, V987P), known as the “S-2P” modification [34]. A previous study demonstrated that VLPs assembled with NDV NP, M proteins, and the extracellular domain of the S-2P-modified S protein fused to the F protein could induce high neutralizing antibody titers in immunized mice [35]. Furthermore, the substitution of the S1/S2 cleavage site (RRAR to QQAQ), combined with the S-2P modification, has been shown to enhance the S protein’s proteolytic resistance and structural stability, resulting in the “S-3Q-2P” modification [36]. Despite these advances, the application of the S-3Q-2P modification in NDV VLP vaccines has not yet been reported.

Based on this, we developed a recombinant NDV-VLP vaccine displaying the modified S-3Q-2P protein, namely NDV-S3Q2P-VLP. Using a prime-boost vaccination regimen in animal studies, the vaccine induced a strong humoral immune response in mice, as evidenced by elevated levels of S-protein-specific IgG and neutralizing antibodies against a SARS-CoV-2 pseudovirus. Additionally, the vaccine effectively stimulated cellular immunity. These findings highlight NDV-S3Q2P-VLP as a promising candidate for future clinical trials in the fight against COVID-19.

## 2. Materials and Methods

### 2.1. Ethics Statement

This study was conducted in strict adherence to the guidelines set forth in the Guide for the Care and Use of Laboratory Animals issued by the Ministry of Science and Technology of the People’s Republic of China. The animal experiment protocols were approved by the Jiangsu Administrative Committee for Laboratory Animals (approval number: SYXK-SU-2022-0040, 26 March 2021) and followed the welfare and ethical standards established by the committee.

### 2.2. Construction of Recombinant Baculovirus Plasmids

The SARS-CoV-2 S-3Q-2P gene used in this study was derived from the wild-type S gene (GenBank accession number: MN908947, positions 21563 to 25384) by mutating the cleavage site ^682^RRAR^685^ to ^682^QQAQ^685^ and introducing two proline substitutions (K986P, V987P). The S-3Q-2P gene was synthesized and cloned into the pUC57 vector (TransGen, Nanjing, China) by GenScript Biotech Corporation (Nanjing, China), and the plasmid was named pUC57-S-3Q-2P. The S-3Q-2P gene ectodomain (residues 1–1208) was amplified from the pUC57-S3Q2P plasmid using primers pVLS3Q2P-F/pVLS3Q2P-R.

The AI4 strain of the NDV/JS-5-05-GO genotype VII (GenBank accession number: JN631747) was isolated and preserved in our laboratory. Using the full-length clone plasmid of the AI4 strain as a template, three sets of primers (pVLM-F/pVLM-R, pVLNP-F/pVLNP-R, and pVLFtmct-F/pVLFtmct-R) were used to amplify NDV’s M gene, NP gene, and TM and CT domains of F gene (Ftmct, residues 501–533), respectively.

The insect cell expression vector pVL1393 (HonorGene, Changsha, China) was digested with *BamH* I and *EcoR* I (TransGen, China). The digested pVL1393 was then recombined with the S-3Q-2P and NDV’s Ftmct fragments using the ClonExpress Ultra One Step Cloning Kit (Vazyme, Nanjing, China) according to the manufacturer’s instructions to construct transfer plasmid pVL-S3Q2P-Ftmct. Similarly, the NDV’s M and NP genes were lined by a self-cleaving peptide (P2A) and recombined with the digested pVL1393 to construct transfer plasmid pVL-M-P2A-NP.

All primers were synthesized by Tsingke Biotechnology Corporation (Nanjing, China), and the sequences are listed in Table 1.

### 2.3. Generation of Recombinant Baculoviruses (rBVs)

rBVs were generated as described in our previous studies [37,38]. Briefly, Sf9 insect cells were transfected with 500 ng of each transfer plasmid and 100 ng of linearized Autographa californica multiple nucleopolyhedrovirus (AcMNPV) genomic DNA using the TransIntro EL Transfection Reagent (TransGen, China), following the manufacturer’s instructions. The transfected Sf9 cells were cultured in SF900III SFM medium supplemented with 5% fetal bovine serum (FBS, Invitrogen, Carlsbad, CA, USA) at 27 °C. Seventy-two hours post-transfection (hpt), the supernatant was collected by centrifugation at 3000 rpm for 10 min. The presence of rBVs in the supernatant was confirmed via an indirect immunofluorescence assay (IFA) and Western blot. The rescued rBVs, designated as rBV-M-P2A-NP and rBV-S3Q2P-Ftmct, were purified through three rounds of plaque selection in Sf9 cells and passaged in suspension culture for six generations.

### 2.4. IFA

To evaluate the expression of indicated proteins in the rBVs, Sf9 cells were infected with the respective rBVs at an MOI of 1, following our previously established protocols [37,38]. At 96 h post-infection (hpi), the cells were fixed and permeabilized with ice-cold methanol for 10 min −20 °C. Then, the cells were incubated with either NDV-positive chicken serum (1:200, prepared by our laboratory), a rabbit monoclonal antibody (mAb) against the S protein (1:1000, GeneTex, China), or a mouse mAb-targeting baculovirus gp64 (1:1000, Santa Cruz, Dallas, TX, USA) overnight at 4 °C. Following washing, Alexa Fluor 488-conjugated goat anti-chicken IgY, Alexa Fluor 488-conjugated goat anti-mouse IgG (1:2000, TransGen, China), or Alexa Fluor 594-conjugated goat anti-rabbit IgG (1:2000, TransGen, China) was applied as the secondary antibody for 1 h at 37 °C. Fluorescence signals were captured using a Leica fluorescence microscope (Leica, Weztlar, Germany).

For determining the 50% tissue-culture-infectious dose (TCID_50_), the Sf9 cells were seeded in 96-well plates and infected with serial dilutions of the rBVs. At 96 hpi, the level of infection was determined by detecting gp64 expression via IFA. TCID_50_ values were calculated using a standard protocol [39].

### 2.5. VLP Production, Characterization, and Purification

The Sf9 cells were infected with rBV-M-P2A-NP or rBV-S3Q2P-Ftmct at an MOI of 1. At 72 hpi, the supernatant was collected, and cell debris was removed using centrifugation at 3000 rpm for 10 min. The VLPs in the supernatant were concentrated with Amicon Ultra 15 mL Centrifugal Filters (Merck, San Jose, CA, USA). For purification, the concentrated VLPs were subjected to sucrose gradient ultracentrifugation. Samples were layered on top of a sucrose gradient (30%, 40%, 50%, 60% *w*/*v*) and ultracentrifuged at 100,000× *g* for 2 h at 4 °C. After centrifugation, fractions in the intermediate layer were carefully collected. To remove the sucrose, the fractions were centrifuged again at 100,000× *g* for 1.5 h at 4 °C, and the resulting pellet was resuspended in a small volume of PBS and stored at 4 °C. The concentration of VLP protein was determined using a BCA protein assay kit (Beyotime, Nanjing, China). For visualization, the purified VLPs were negatively stained with 1% phosphotungstic acid, dried by aspiration, and observed using a Tecnai 12 transmission electron microscope (Philips, Auckland, The Netherlands) at 100 kV. The generated VLPs were designated as NDV-S3Q2P-VLPs.

### 2.6. Western Blot

To evaluate foreign protein expression in rBVs, the Sf9 cells were infected with rBV-M-P2A-NP or rBV-S3Q2P-Ftmct at an MOI of 1. At 72 hpi, the cells were washed with cold PBS and lysed in RIPA buffer containing protease and phosphatase inhibitors (Beyotime, China). Protein concentrations were determined using a BCA protein assay kit (Beyotime, China). Equal amounts of protein (10 μg) were resolved on 10% SDS-PAGE gels and transferred to PVDF membranes (Millipore, San Diego, CA, USA). Membranes were blocked with 5% skimmed milk in TBST, followed by incubation with primary antibodies at 4 °C overnight. HRP-conjugated secondary antibodies were applied for 1 h at 37 °C. Protein detection was carried out using an ECL system (TransGen, China). To verify successful VLP assembly, 10 μg of NDV-S3Q2P-VLPs was analyzed via Western blot to detect NDV NP, M, and SARS-CoV-2 S proteins, as described above.

NDV-positive chicken serum was produced by our laboratory. The rabbit mAb against the SARS-CoV-2 S protein was sourced from GeneTex (China). The mouse mAb against baculovirus gp64 was obtained from Santa Cruz (USA), and the mouse mAb against β-actin, along with HRP-conjugated goat anti-rabbit, anti-mouse, and anti-chicken secondary antibodies, were purchased from TransGen (China).

### 2.7. Immunization Study of BALB/c Mice

To prepare the VLP vaccine, purified NDV-S3Q2P-VLPs were combined with Montanide ISA 71 VG adjuvant at a 1:1 volume ratio and homogenized vigorously at 6000 r/min for 30 min to ensure thorough emulsification. To assess the immunogenicity of NDV-S3Q2P-VLPs, six-week-old BALB/c mice (n = 6 per group) received twice intramuscular injections of the VLP vaccine at doses of 2 μg, 10 μg, and 20 μg. Mice injected with 0.1 mL PBS served as controls. The initial injection was administered on day 0, followed by a booster on day 14. Blood samples were collected from the tail vein on days 14 and 28 and centrifuged at 4000 rpm for 5 min to isolate serum, which was subsequently used for the detection of S protein specific IgG antibody titers and neutralizing titers.

### 2.8. Determination of the S Protein-Specific IgG, IgG1, and IgG2a Antibody Titers

S-protein-specific IgG, IgG1, and IgG2a antibody titers were measured using an enzyme-linked immunosorbent assay (ELISA). Briefly, 200 ng of purified S protein (Sino Biological, Beijing, China) was coated onto a 96-well flat-bottom microplate and incubated overnight at 4 °C. For S-protein-specific IgG detection, serial dilutions of mouse serum were added to the coated wells and incubated at 37 °C for 1 h. For IgG1 and IgG2a detection, a 1:40 dilution for IgG1 and a 1:400 dilution for IgG2a of each serum sample were added to the appropriate wells and incubated at 37 °C for 1 h. After three washes with TBST, 100 μL of HRP-conjugated goat anti-mouse IgG (1:4000, TransGen, China), anti-mouse IgG1 (1:4000, Santa Cruz, USA), or anti-mouse IgG2a (1:4000, Santa Cruz, USA) was added to each well and incubated at 37 °C for 1 h. Following another round of washes, 100 μL of 3,3′,5,5′-tetramethylbenzidine (TMB) substrate was added, and the plate was incubated for 15 min at room temperature. The reaction was then stopped by adding 50 μL of 2 M H_2_SO_4_, and absorbance was measured at 450 nm using a microplate reader (BioTek, Winooski, VT, USA).

### 2.9. Construction of ACE2 Overexpressing 293T Cell Line

To generate a stable 293T cell line expressing human Angiotensin-converting enzyme 2 (ACE2), the HIV-1 three-plasmid lentiviral packaging system was employed, as previously described [40]. Briefly, lentiviral vectors carrying the human ACE2 gene (pLenti-hACE2-PURO), packaging plasmid (psPAX2), and envelope plasmid (pMD2.G) were obtained from Addgene (Seattle, WA, USA). 293T cells were cultured in Dulbecco’s modified Eagle’s medium (DMEM, Thermo, Waltham, MA, USA) supplemented with 10% FBS at 37 °C and 5% CO_2_ until they reached 70–80% confluency. The cells were then co-transfected with 10 μg of pLenti-hACE2-PURO, 7.5 μg of psPAX2, and 5 μg of pMD2.G using Lipofectamine 2000 (Invitrogen, USA), according to the manufacturer’s protocol. At 48 hpt, the medium was replaced with fresh DMEM. Lentiviral particles were collected at 72 hpt, centrifuged at 3000 rpm for 10 min, and filtered through a 0.45 μm filter. For transduction, 293T cells were seeded in 6-well plates, infected with the filtered lentiviral supernatant, and supplemented with 9 μg/mL polybrene (MCE, Shanghai, China). After 24 h, the medium was replaced. Forty-eight hours later, Puromycin at a final concentrate of 8 μg/mL (MCE, China) was added to select stably transduced cells for one week. Stable ACE2 expression was confirmed via Western blot, using anti-ACE2 antibodies (Santa Cruz, USA). The identified cell line was named as 293T-hACE2.

### 2.10. Packaging and Characterization of SARS-CoV-2 Pseudovirus

The S gene was amplified from pUC57-S3Q2P using the following primers: S-F: 5′-CAAGCTGGCTAGCGTTTAAACTTAATGTTTGTTTTTCTTGTTTT-3′ S-R: 5′-CGGGTTTAAACGGGCCCTCTAGTTAGCAGCAGGATCCACAAGA-3′. The amplified S gene was cloned into the *EcoR* I and *Xba* I sites of the pCDNA3.1 vector (Beyotime, China) to construct pcDNA3.1-SARS2-S. For SARS-CoV-2 pseudovirus packaging, pcDNA3.1-SARS2-S, along with the packaging plasmid psPAX2 and the transfer plasmid pLV-Luciferase-GFP, was co-transfected into 293T cells using Lipofectamine 2000 (Invitrogen, USA). Similarly, the pMD2.G plasmid, expressing the vesicular stomatitis virus Glycoprotein protein (VSV-G), was also co-transfected with psPAX2 and pLV-Luciferase-GFP into 293T cells to generate VSV-G pseudovirus. Pseudovirus-containing supernatant was collected at 72 hpt, centrifuged at 3000 rpm for 10 min, and filtered through a 0.45 μm filter.

To verify the infectivity of the SARS-CoV-2 pseudovirus, the virus was diluted in DMEM containing 2% FBS and added to 96-well plates seeded with either 293T-hACE2 cells or wild-type 293T cells. VSV-G pseudovirus was similarly treated as a control. At 48 hpi, GFP expression was observed under a fluorescence microscope, and luciferase activity was measured using the Luciferase Assay System (Promega, Madison, WI, USA) to confirm pseudovirus infectivity.

### 2.11. Pseudovirus Neutralization Assay

To assess neutralizing antibody levels in mouse serum at two weeks post-boost immunization, 293T-hACE2 cells were seeded in 96-well plates (5 × 10^4^ cells/well) and incubated for 12 h at 37 °C. Serum samples were heat-inactivated (56 °C, 30 min) and diluted in serum-free DMEM, starting at 1:10 and serially diluted 2-fold. For the neutralization assay, 50 μL of diluted serum was mixed with 30 μL DMEM with 2% FBS and 20 μL of SARS-CoV-2 pseudovirus, and incubated at 37 °C for 1h. The mixture was then added to the 293T-hACE2 cells. After 12 h, 100 μL of 10% FBS DMEM was added, and incubation continued for 48h. Control wells contained SARS-CoV-2 pseudovirus without serum. Luciferase activity was measured using the Luciferase Assay System (Promega, USA) according to the manufacturer’s instructions. The 50% inhibitory concentration (IC50) was determined based on the serum dilution that resulted in a 50% reduction in luciferase activity.

### 2.12. Analysis of CD4+ and CD8+ T Cells in Lung and Spleen by Flow Cytometry

At 2 weeks post-boost immunization, lung and spleen tissues were harvested for single-cell suspension preparation. The lung tissue was minced and digested with RPMI-1640 medium (Gibco, Waltham, MA, USA) containing 0.5 mg/mL collagenase IV (Sigma-Aldrich, St. Louis, MO, USA) and 0.05 mg/mL DNase I (Sigma-Aldrich, USA) at 37 °C for 30–45 min, followed by filtration through a 70 μm cell strainer (Falcon, Corona, CA, USA). Red blood cells were lysed using ACK lysis buffer (Gibco, USA) and the cells were washed with RPMI-1640 medium (Gibco, USA). The spleen was carefully removed, placed in RPMI-1640 medium (Gibco, USA) on ice, and then gently mashed through a 70 μm cell strainer (Falcon, USA) to create a single-cell suspension. The suspension was washed with RPMI-1640 medium and red blood cells were lysed using ACK lysis buffer (Gibco, USA) for 2–3 min at room temperature. The resulting cell suspension was filtered through a 70 μm strainer (Falcon, USA) and washed twice with RPMI-1640 medium to remove debris. The resulting cells were adjusted to 1 × 10^6^ cells/mL, blocked with anti-mouse CD16/32 antibody (BD Biosciences, Franklin Lakes, NJ, USA), and stained with mouse anti-CD4-APC (BD Biosciences, USA) and anti-CD8-FITC (BD Biosciences, USA) antibodies for 30 min at 4 °C. After washing, the cells were analyzed by BD FACS Aria III flow cytometer (BD Biosciences, USA).

### 2.13. Evaluation of Cellular Immune Response by qRT-PCR

To evaluate the cellular immune response induced by NDV-S3Q2P-VLPs, the mice were euthanized two weeks post-boost immunization, and kidney, liver, spleen, and lung samples were collected from each group. Tissue homogenates were prepared, and the total RNA was extracted using the EasyPure RNA kit (TransGen, China) following the manufacturer’s protocol. cDNA synthesis and qRT-PCR were conducted using the TransScript Green One-Step qRT-PCR Super Mix (TransGen, China) on a LightCycler 480 system (Roche, Basel, Switzerland). Primer sequences for the qRT-PCR are listed in Table 2. β-actin served as the internal control, and relative mRNA expression levels were determined using the 2^−ΔΔ*C*t^ method.

### 2.14. Statistical Analysis

Statistical significance was determined using one-way or two-way ANOVA in GraphPad Prism (GraphPad Software, V8.0, San Diego, CA, USA). A *p*-value of less than 0.05 was considered statistically significant. The significance levels were denoted as follows: NS (not significant), * *p* < 0.05, ** *p* < 0.01, *** *p* < 0.001, **** *p* < 0.0001.

## 3. Results

### 3.1. Generation and Characterization of rBVs

**Figure 1:**
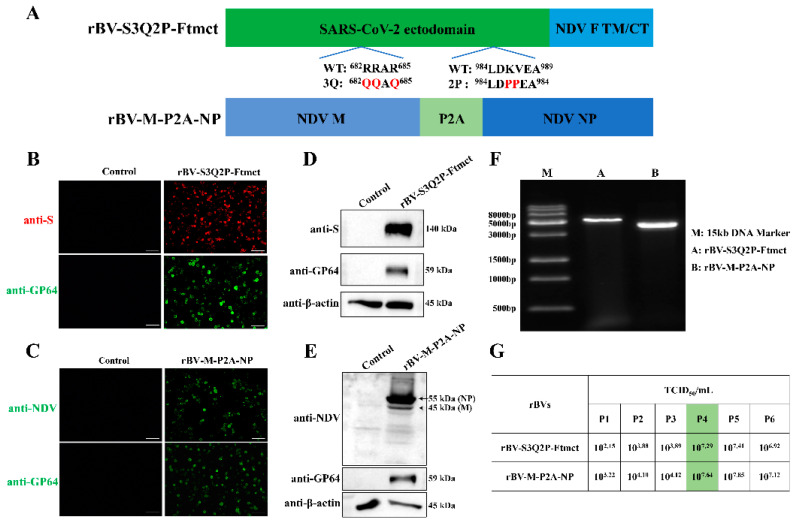
Construction and characterization of recombinant baculoviruses (rBVs). (**A**) Schematic diagram of the rBV constructs. rBV-S3Q2P-Ftmct includes the ectodomain of the SARS-CoV-2 S protein fused to the transmembrane and cytoplasmic tail (TM/CT) domains of the NDV F protein, with the S1/S2 cleavage site (RRAR) mutated to QQAQ and two proline substitutions (K986P, V987P) for stabilization. rBV-M-P2A-NP includes the NDV M and NP proteins, linked via the P2A sequence. (**B**,**C**) Immunofluorescence assay (IFA) of Sf9 cells infected with rBV-S3Q2P-Ftmct (**B**) and rBV-M-P2A-NP (**C**). Cells were stained with anti-S (red) or anti-NDV (green) antibodies, along with anti-GP64 (green) as a baculovirus marker. Scale bars,100 μm. (**D**,**E**) Western blot analysis of Sf9 cells infected with rBV-S3Q2P-Ftmct (**D**) and rBV-M-P2A-NP (**E**). Proteins were detected using antibodies against SARS-CoV-2 S protein, NDV NP, M, and GP64, and β-actin was used as a control. (**F**) PCR confirmation of the rBV constructs. The presence of the expected gene fragments for rBV-S3Q2P-Ftmct (lane A) and rBV-M-P2A-NP (lane B) was confirmed. (**G**) Virus titration results (TCID_50_/mL) of the rBVs across passages (P1–P6).

To construct rBVs, the ectodomain of the SARS-CoV-2 S protein, fused with the TM and CT domains of the NDV F protein, was cloned into the insect cell expression vector pVL1393. Additionally, the NDV M and NP genes, linked via the P2A sequence, were also cloned into pVL1393 vector. The construction scheme is illustrated in Figure 1A. The successful generation of rBVs was confirmed through IFA, Western blot, and PCR. As shown in Figure 1B,C, Sf9 cells infected with rBV-S3Q2P-Ftmct or rBV-M-P2A-NP exhibited strong fluorescence signals when stained with specific antibodies, whereas uninfected cells displayed no fluorescence. Western blot analysis revealed that rBV-S3Q2P-Ftmct-infected Sf9 cells expressed the SARS-CoV-2 S protein at approximately 140 kDa, along with the baculovirus marker protein GP64 (Figure 1D). Similarly, rBV-M-P2A-NP-infected cells expressed both NDV NP and M proteins, as detected using NDV-positive chicken serum (Figure 1E). In addition, the PCR analysis confirmed the presence of the expected gene fragments in the rBV genomes (Figure 1F). These results indicate the successful construction of rBV-S3Q2P-Ftmct and rBV-M-P2A-NP.

Both recombinant baculoviruses were serially passaged in Sf9 cells, and the virus titers were measured at each passage. As shown in Figure 1G, virus titers stabilized at approximately 10^7.0^ TCID_50_/mL by the fourth passage. Consequently, the fourth passage of rBVs was selected for subsequent VLP assembly experiments.

### 3.2. Production and Characterization of NDV-S3Q2P-VLP

**Figure 2:**
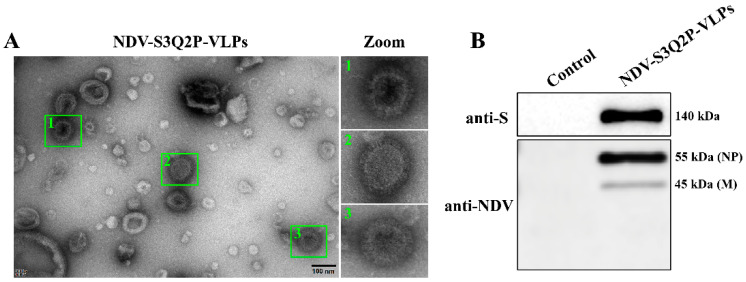
Production and characterization of NDV-S3Q2P-VLPs. (**A**) Transmission electron microscopy (TEM) image of NDV-S3Q2P-VLPs. Sf9 cells were co-infected with rBV-S3Q2P-Ftmct and rBV-M-P2A-NP, and VLPs were purified at 72 hpi. VLPs were negatively stained and visualized under a transmission electron microscope. The zoomed-in sections (1, 2, and 3) provide magnified views of individual VLPs. Scale bars, 100 nm. (**B**) Western blot analysis of NDV-S3Q2P-VLPs. Purified VLPs were lysed and subjected to SDS-PAGE, followed by immunoblot with anti-SARS-CoV-2 S protein and anti-NDV antibodies. S protein (140 kDa), NDV NP (55 kDa), and M (45 kDa) were detected.

To assemble NDV-S3Q2P-VLPs, the Sf9 cells were co-infected with rBV-S3Q2P-Ftmct and rBV-M-P2A-NP at an MOI of 1. The successful assembly of VLPs was confirmed through TEM and Western blot analysis. As illustrated in Figure 2A, the TEM revealed spherical particles with a size of approximately 100 nm, which is consistent with the expected morphology of VLPs. These particles displayed a compact structure similar to native viruses, suggesting the successful assembly of NDV-S3Q2P-VLPs. Furthermore, the Western blot analysis verified the incorporation of the SARS-CoV-2 S protein and NDV M and NP proteins into the VLPs. The S3Q2P-VLPs exhibited strong bands at approximately 140 kDa, 55 kDa, and 45 kDa, corresponding to the S, M, and NP protein, respectively (Figure 2B). These results collectively confirm the successful production and assembly of NDV-S3Q2P-VLPs.

### 3.3. Validation of 293T-hACE2 Cell Line and SARS-CoV-2 Pseudovirus

**Figure 3:**
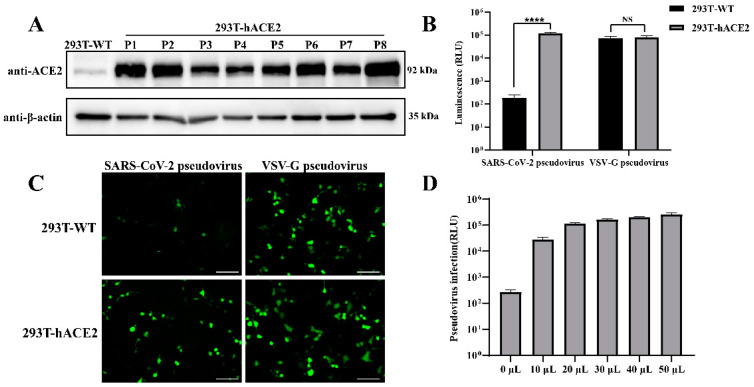
Validation of 293T-hACE2 cell line and SARS-CoV-2 pseudovirus infectivity: (**A**) Western blot analysis of ACE2 expression in 293T wild-type (WT) and 293T-hACE2 cells from passages 1 to 8: Cells were lysed and subjected to SDS-PAGE, followed by immunoblotting using anti-ACE2 and anti-β-actin antibodies. (**B**) Luciferase assay to assess pseudovirus infectivity: 293T-WT and 293T-hACE2 cells were infected with SARS-CoV-2 pseudovirus or VSV-G pseudovirus, and luciferase activity was measured after 48 h to evaluate infection efficiency. (**C**) Fluorescence microscopy to visualize pseudovirus infection: 293T-WT and 293T-hACE2 cells were infected with SARS-CoV-2 pseudovirus or VSV-G pseudovirus, and GFP fluorescence was observed at 48 hpi. Scale bars, 100 μm. (**D**) Pseudovirus infection dose–response experiment: 293T-hACE2 cells were infected with increasing volumes of SARS-CoV-2 pseudovirus (10 μL, 20 μL, 30 μL, 40 μL, and 50 μL), and luciferase activity was measured at 48hpi to determine the optimal pseudovirus dose. NS means not significant, **** *p* < 0.0001.

A stable 293T cell line expressing human ACE2 was generated using the HIV-1 three-plasmid lentiviral packaging system. To verify the stable expression of ACE2, 293T-hACE2 cells from passages 1 to 8 were analyzed using Western blot. As shown in Figure 3A, all generations of 293T-hACE2 cells consistently expressed high levels of ACE2 protein, whereas wild-type 293T cells showed minimal expression. These results confirm the successful construction of the stable 293T-hACE2 cell line.

SARS-CoV-2 pseudovirus was generated using the same lentiviral system. To assess its infectivity, 293T-hACE2 cells and 293T wild-type cells were infected with the packaged SARS-CoV-2 pseudovirus or VSV-G pseudovirus, and luciferase activity and GFP fluorescence were measured at 48 hpi. As shown in Figure 3B, the SARS-CoV-2 pseudovirus exhibited significantly higher luciferase activity in 293T-hACE2 cells compared to 293T wild-type cells, whereas the control VSV-G pseudovirus showed high luciferase activity in both cell lines. Additionally, a fluorescence analysis (Figure 3C) demonstrated strong GFP expression in 293T-hACE2 cells infected with SARS-CoV-2 pseudovirus, while minimal fluorescence was observed in wild-type 293T cells. In contrast, the VSV-G pseudovirus showed strong GFP signals in both cell lines. These results confirm the successful generation of infectious SARS-CoV-2 pseudovirus capable of specifically infecting 293T-hACE2 cells.

To determine the optimal infection dose of SARS-CoV-2 pseudovirus for subsequent neutralization assays, 293T-hACE2 cells were infected with increasing volumes of SARS-CoV-2 pseudovirus (10 μL, 20 μL, 30 μL, 40 μL, and 50 μL), and luciferase activity was measured after 48 h. As shown in Figure 3D, the luciferase activity reached over 1 × 10^5^ with 20 μL of pseudovirus, and further increases in pseudovirus volume resulted in only marginal increases. Therefore, 20 μL of SARS-CoV-2 pseudovirus was selected as the optimal dose for subsequent neutralization assays.

### 3.4. NDV-S3Q2P-VLPs Immunization Elicits High Titers of S Protein-Specific IgG, IgG1, and IgG2a Antibodies in Mice

**Figure 4:**
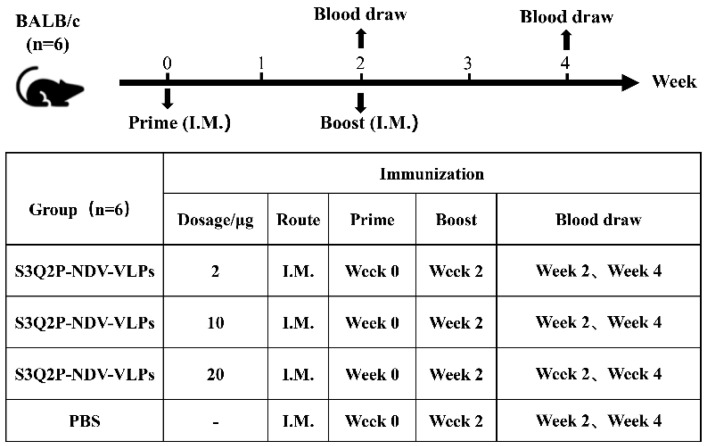
Immunization schedule and groups: BALB/c mice (n = 6 per group) were immunized with different doses of NDV-S3Q2P-VLPs (2 μg, 10 μg, and 20 μg) or PBS as a control. Mice were immunized intramuscularly (I.M.) with a prime dose at week 0 and a boost dose at week 2. Blood samples were collected at week 2 and week 4 to assess immune responses.

To assess the immunogenicity of NDV-S3Q2P-VLPs, BALB/c mice (n = 6 per group) were immunized intramuscularly (I.M.) with 2 μg, 10 μg, or 20 μg of NDV-S3Q2P-VLPs, while a control group received PBS. As outlined in Figure 4, mice received a prime immunization at week 0 and a booster immunization at week 2. Blood samples were collected at weeks 2 and 4 to measure antibody responses.

**Figure 5:**
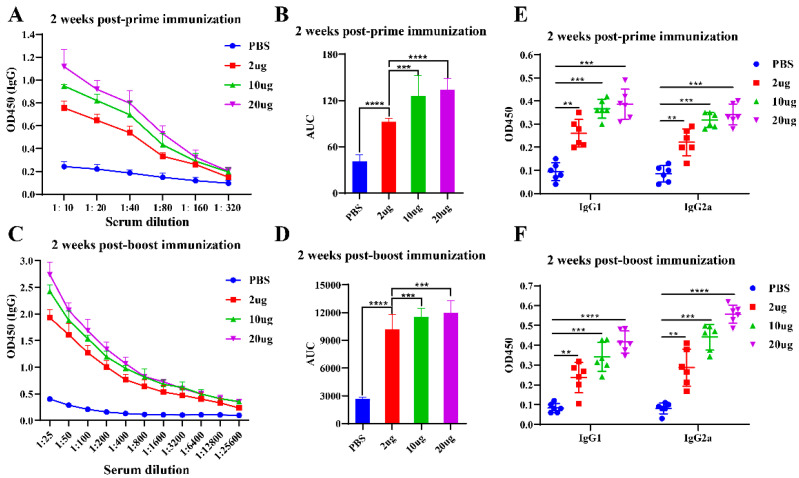
S-protein-specific IgG, IgG1, and IgG2a antibody responses induced by NDV-S3Q2P-VLPs immunization. (**A**) ELISA analysis of S-protein-specific IgG antibody titers at 2 weeks post-prime immunization. BALB/c mice were immunized with NDV-S3Q2P-VLPs at doses of 2 μg, 10 μg, or 20 μg, with PBS as the control. Serum samples were collected and serially diluted (1:10 to 1:320) to assess IgG titers. (**B**) Area under the curve (AUC) analysis of S-protein-specific IgG titers at 2 weeks post-prime immunization: AUC values were calculated to quantify the antibody responses across different doses. (**C**) ELISA analysis of S-protein-specific IgG antibody titers at 2 weeks post-boost immunization: serum samples were serially diluted (1:25 to 1:25,600) to measure the increase in IgG titers following the booster dose. (**D**) AUC analysis of S-protein-specific IgG titers at 2 weeks post-boost immunization: AUC values were calculated to evaluate the dose-dependent response after the boost. (**E**) ELISA analysis of S-protein-specific IgG1 and IgG2a antibody titers at 2 weeks post-prime immunization: serum samples were diluted at 1:40. (**F**) ELISA analysis of S-protein-specific IgG1 and IgG2a antibody titers at 2 weeks post-boost immunization: serum samples were diluted at 1:400. Statistical analyses were performed using one-way ANOVA for (**B**,**D**) and two-way ANOVA for (**E**,**F**). Data are presented as means ± standard deviation (SD). ** *p* < 0.01, *** *p* < 0.001, **** *p* < 0.0001.

S-protein-specific IgG titers were measured using ELISA. As shown in Figure 5A, all VLP-immunized groups exhibited significantly higher IgG titers compared to the PBS group at 2 weeks post-prime. Antibody titers increased with dose, with the 20 μg group showing the highest titers. An area under the curve (AUC) analysis confirmed a dose-dependent antibody response (Figure 5B). At 2 weeks post-boost, IgG titers continued to rise in all immunized groups (Figure 5C), and an AUC analysis (Figure 5D) also demonstrated robust IgG responses in all VLP-immunized groups, significantly higher than in the PBS group. These results suggest that NDV-S3Q2P-VLPs elicit a potent, dose-dependent S-protein-specific IgG response, which is further enhanced following the booster immunization.

Different IgG antibody isotypes were used to evaluate the type of T helper (Th) response [41]. IgG1 is typically associated with Th2-type immune responses, which are generally characterized by humoral immunity, while IgG2a is more strongly linked to Th1-type immune responses, which are characterized by cell-mediated immunity [42]. To further characterize the IgG subclasses induced by the VLP vaccine, S-protein-specific IgG1 and IgG2a titers were measured by ELISA. As shown in Figure 5E, at 2 weeks post-prime, all VLP-immunized groups induced significant upregulation of both IgG1 and IgG2a compared to the PBS group. The IgG1/IgG2a ratio was approximately 1.1 (2 μg), 1.2 (10 μg), and 1.2 (20 μg). Similarly, at 2 weeks post-boost, all VLP vaccine doses significantly increased IgG1 and IgG2a levels, with the IgG1/IgG2a ratio at 0.9 (2 μg), 0.8 (10 μg), and 0.7 (20 μg). These results indicate that immunization with NDV-S3Q2P-VLPs stimulates both arms of the immune system, eliciting a well-balanced Th1/Th2 immune response.

### 3.5. NDV-S3Q2P-VLP Immunization Triggers Potent Neutralizing Antibody Responses Against SARS-CoV-2 Pseudovirus

**Figure 6:**
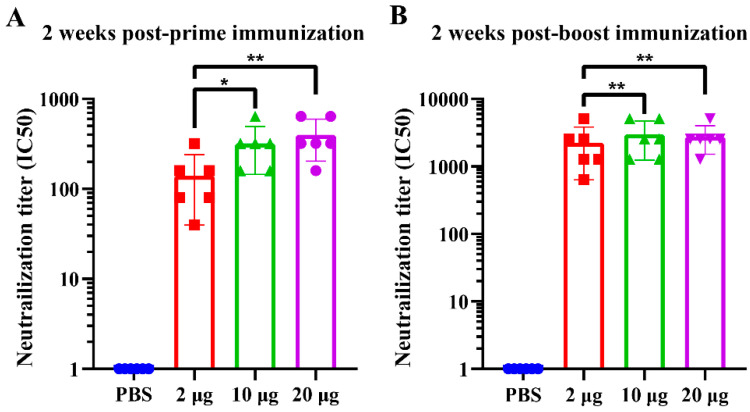
Neutralizing-antibody titers against SARS-CoV-2 pseudovirus induced by NDV-S3Q2P-VLPs immunization: (**A**) Neutralizing-antibody titers (IC50) at 2 weeks post-prime immunization. BALB/c mice were immunized with NDV-S3Q2P-VLPs at doses of 2 μg, 10 μg, or 20 μg, with PBS as the control. Serum samples were collected and tested for neutralization of SARS-CoV-2 pseudovirus. IC50 values represent the dilution at which 50% neutralization was achieved. (**B**) Neutralizing antibody titers (IC50) at 2 weeks post-boost immunization: serum samples were collected and tested for neutralization activity against SARS-CoV-2 pseudovirus following the booster dose. Statistical analyses were performed using one-way ANOVA, and data are presented as means ± SD. * *p* < 0.05, ** *p* < 0.01.

To assess the ability of NDV-S3Q2P-VLPs to induce neutralizing antibodies, serum samples from immunized mice were evaluated for neutralizing activity against SARS-CoV-2 pseudovirus. Neutralization titers (IC50) were measured 2 weeks post-prime and post-boost immunizations. As shown in Figure 6A, at 2 weeks post-prime, all NDV-S3Q2P-VLP immunized groups exhibited significantly higher neutralizing antibody titers compared to the PBS control group. Following the booster immunization (Figure 6B), neutralizing-antibody titers increased dramatically across all VLP-immunized groups, with the 10 μg and 20 μg dose groups demonstrating the highest neutralizing activity. These results indicate that NDV-S3Q2P-VLPs elicit robust neutralizing-antibody responses in a dose-dependent manner, with all doses providing strong immune response.

### 3.6. NDV-S3Q2P-VLP Immunization Promotes Robust T Cell Immunity in Mice

**Figure 7:**
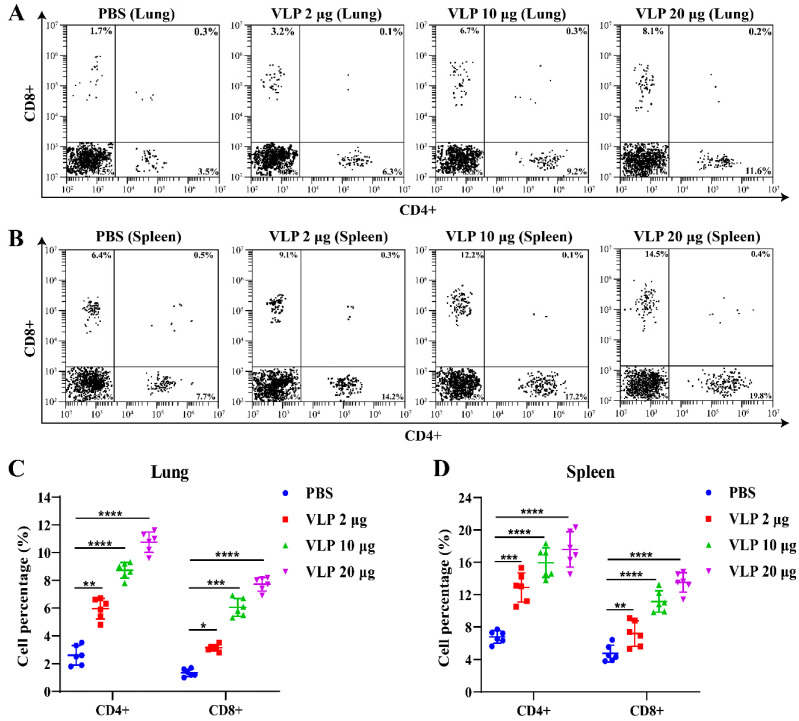
NDV-S3Q2P-VLPs induced CD4+ and CD8+ T cell responses in lung and spleen two weeks after booster immunization: Lung and spleen single-cell suspensions were isolated from mice at 2 weeks post-boost immunization with different doses of NDV-S3Q2P-VLPs or PBS control. The cells were stained with mouse anti-CD4 and anti-CD8 antibodies to evaluate T cell populations. (**A**) Flow cytometry analysis of CD4+ and CD8+ T cell populations in the lung. (**B**) Flow cytometry analysis of CD4+ and CD8+ T cell populations in the spleen. (**C**) Proportional analysis of CD4+ and CD8+ T cells in the lung. (**D**) Proportional analysis of CD4+ and CD8+ T cells in the spleen. Data were analyzed with two-way ANOVA. * *p* < 0.05, ** *p* < 0.01, *** *p* < 0.001, **** *p* < 0.0001.

To evaluate the T cell responses induced by NDV-S3Q2P-VLPs, flow cytometry analysis was performed to assess the CD4+ and CD8+ T cell populations in the lung and spleen of immunized mice, 2 weeks after immunization with different doses of the vaccine. The results showed that all VLP vaccine doses induced a significantly higher number of CD4+ and CD8+ T cells in the lung compared to the PBS control group, with the 20 μg dose showing the most pronounced difference (Figure 7A,C). Similarly, CD4+ and CD8+ T cells were also significantly increased in the spleen of VLP vaccine-immunized mice (Figure 7B,D). In addition, the upregulation of CD4+ and CD8+ T cells in the spleen was more pronounced than that observed in the lung. These findings provide strong evidence of the NDV-S3Q2P-VLP vaccine’s ability to activate both CD4+ and CD8+ T cell responses in the lung and spleen, highlighting the vaccine’s potential to stimulate T cell-mediated immunity.

**Figure 8:**
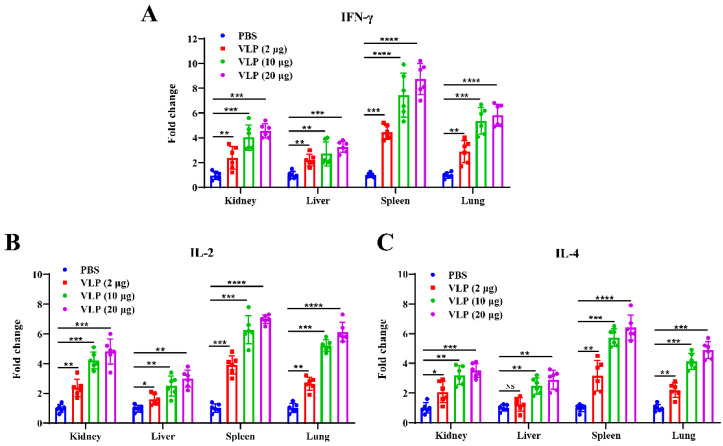
Cytokine expression levels in tissues from mice immunized with NDV-S3Q2P-VLPs. Total RNA was extracted from kidney, liver, spleen, and lung tissues of NDV-S3Q2P-VLP-immunized mice or PBS control at 2 weeks post-boost immunization. qRT-PCR was used to measure the expression levels of IFN-γ, IL-2, and IL-4 in these tissues. (**A**) Fold change in IFN-γ expression in kidney, liver, spleen, and lung tissues. (**B**) Fold change in IL-2 expression in kidney, liver, spleen, and lung tissues. (**C**) Fold change in IL-4 expression in kidney, liver, spleen, and lung tissues. Cytokine expression was normalized to β-actin, and relative expression was calculated using the 2^−ΔΔCt^ method. Statistical analyses were performed using two-way ANOVA. NS means not significant, * *p* < 0.05, ** *p* < 0.01, *** *p* < 0.001, **** *p* < 0.0001.

Next, to further characterize the type of Th response induced, qRT-PCR was used to measure the expression levels of key cytokines, including IFN-γ, IL-2, and IL-4, in kidney, liver, spleen, and lung tissues of all immunized mice at 2 weeks post-boost immunization. As shown in Figure 8A, the expression of IFN-γ, a key marker of Th1-type immune response [43], was significantly upregulated in all the tissues of the NDV-S3Q2P-VLP-immunized group compared to the PBS control group. The highest fold change was observed in the spleen, indicating a strong systemic Th1 response. Similarly, IL-2 expression (Figure 8B), another Th1 cytokine [43], was significantly higher in the spleen, lung, and liver of the VLP-immunized mice, compared to controls. In addition, the Th2 cytokine IL-4 [44] was also markedly upregulated in the VLP-immunized mice, particularly in the spleen, lung, and kidney tissues (Figure 8C). Notably, the immune response was dose-dependent: the high-dose vaccine group (20 μg) exhibited significantly higher levels of IFN-γ, IL-2, and IL-4 compared to the low-dose group (2 μg) across all tissues, suggesting a dose-dependent enhancement of both Th1 and Th2 immune responses. These results demonstrate that NDV-S3Q2P-VLP post-boost immunization induces a robust T cell-mediated immunity characterized by both Th1 (IFN-γ, IL-2) and Th2 (IL-4) cytokine production.

## 4. Discussion

NDV is an avian virus that has been extensively researched as an oncolytic virus, with a long history of clinical trials for this application [30]. Due to host range restriction, it is not pathogenic in humans, which avoids the issue of preexisting immunity in the population. This study presents the successful development of an NDV-based VLP vaccine, NDV-S3Q2P-VLP, designed to elicit strong humoral and cellular immune responses against SARS-CoV-2. We engineered the S protein with stabilizing modifications, including the S-2P and S-3Q modifications, to enhance immunogenicity and stability. The immunogenicity of this VLP vaccine was evaluated in BALB/c mice, and the results demonstrated that the NDV-S3Q2P-VLP vaccine induced high titers of S-protein-specific IgG, IgG1, and IgG2a antibodies and potent neutralizing antibody titers against SARS-CoV-2 pseudovirus. Furthermore, the vaccine significantly activated CD4+ and CD8+ T cells in both the lungs and spleen and upregulated the transcription of Th1 cytokines (IFN-γ, IL-2) and Th2 cytokine (IL-4).

The immunogenicity of NDV-based SARS-CoV-2 vaccines has been explored in various preclinical studies [45,46,47,48,49]. In the context of viral vector vaccines, previous research has shown that NDV-based SARS-CoV-2 vaccines expressing the full-length S protein, the S1 protein, or the RBD can all elicit significant levels of neutralizing antibodies in animal models. For example, Sun et al. developed an NDV vector expressing the cleavage-deficient SARS-CoV-2 S protein, which induced neutralizing antibody titers ranging from 1:50 to 1:400 in mice and hamsters at 11 days post-boost immunization [48]. However, in this study, the NDV-S3Q2P-VLP vaccine elicited higher neutralizing antibody titers (1:140 to 1:400) as early as two weeks post-primary immunization, and following the booster dose, titers increased significantly, reaching up to 1:2560. Despite potential variability between different batches of experiments, the neutralizing antibody levels induced by NDV-S3Q2P-VLP appear to be higher than those observed with the inactivated NDV-based SARS-CoV-2 vaccine.

Additionally, a previous study of an NDV-based VLP vaccine, S2P-NDVLP, which displayed the prefusion-stabilized S protein, reported high neutralizing titers post-boost, ranging from 2125 to 4552, depending on the dosage [35]. In our study, the VLP vaccine, which incorporated both the S2P and S3Q modifications, induced neutralizing antibody titers comparable to those of the S2P-NDVLP vaccine after booster immunization. While both vaccines showed similar immune responses post-boost, our results at two weeks post-primary immunization demonstrated that the NDV-S3Q2P-VLP vaccine may induce higher neutralizing antibody levels earlier. However, further comparative studies are needed to determine if this early response provides a measurable clinical advantage.

When comparing mRNA vaccines, which have shown neutralizing-antibody titers in the range of 1:500 to 1:5000 in clinical trials [50,51], different dose of NDV-S3Q2P-VLPs (2, 10, and 20 µg) demonstrated neutralization titers in the range of 1:2200–1:2560 at 2 weeks post-boost immunization. Furthermore, while mRNA vaccines such as Pfizer-BioNTech’s BNT162b2 have been shown to rapidly induce high neutralizing-antibody titers after two doses [8], our study indicates that NDV-S3Q2P-VLPs can also generate strong neutralizing activity after the prime-boost regimen, with comparable titers post-boost. These highlight the potential of NDV-based VLPs as a competitive alternative to mRNA vaccines, especially in scenarios where vaccine production and storage logistics are more challenging.

In addition to its humoral immunity, our study showed that NDV-S3Q2P-VLPs also elicited strong cellular immune responses. This is critical for long-term protection against viral infections, as cellular immunity plays a key role in eliminating infected cells and providing broader immunity [52,53]. Previous studies have demonstrated that SARS-CoV-2 VLP vaccines can induce potent T cell responses [54]. Charland et al. reported that a coronavirus VLP vaccine induced significant Th1 (IFN-γ) and Th2 (IL-4) responses after the second immunization. Similarly, our results showed that NDV-S3Q2P-VLP vaccination significantly upregulated IFN-γ, IL-2, and IL-4 in multiple organs, with the spleen exhibiting the highest levels of IFN-γ and IL-2, indicating strong T cell activation in lymphoid tissues. Moreover, our study demonstrated that CD4+ and CD8+ T cells were significantly increased in both the spleen and lung of immunized mice, with the 20 µg dose group showing the highest responses. This robust activation of CD4+ and CD8+ T cells highlights the vaccine’s capacity to stimulate a comprehensive cellular immune response, essential for long-term protection.

Despite the promising results obtained in this study, there are still challenges to address. One concern is that live virus challenge experiments with NDV-S3Q2P-VLPs are strictly regulated in China due to biosafety issues. Our animal biosafety level 3 (BSL-3) laboratory is not authorized to perform SARS-CoV-2 challenge studies, meaning that the challenge protection data for NDV-S3Q2P-VLPs are still pending. Further research in authorized facilities will be necessary to fully evaluate the protective efficacy of NDV-S3Q2P-VLPs against live virus infection, particularly against emerging SARS-CoV-2 variants, such as Delta and Omicron. Another limitation of this study is the vaccination schedule. Despite the two-week interval between doses, we observed that the NDV-VLP-S-3Q2P vaccine successfully induced significant immune responses. However, longer intervals between doses (e.g., four weeks) should be further evaluated to comprehensively assess the optimal immunization schedule for NDV-VLP-S-3Q2P.

In conclusion, the NDV-S3Q2P-VLP vaccine developed in this study demonstrated the ability to induce robust humoral and cellular immune responses, including high levels of IgG, IgG1, IgG2a and neutralizing antibodies, activation of Th1/Th2 cytokines, and strong CD4+ and CD8+ T cell responses. These results support the potential of NDV-S3Q2P-VLP as a promising vaccine for global COVID-19 prevention and control.

## Figures and Tables

**Table 1:** Primers used for construction of recombinant baculovirus plasmid.

Primer Name	Primer Sequence (5′-3′)
pVLS3Q2P-F	CCACCATCGGGCGCGGATCCATGTTTGTTTTTCTTGTT
pVLS3Q2P-R	CTGCTCATACTTTCCAAGTTCTTGGAGATCGATG
pVLFtmct-F	AACTTGGAAAGTATGAGCAGTCTGCTCTCATTACCTAT
pVLFtmct-R	GAGCCACCACAAGAGCATGATCTAGAATTCCGGAGCGG
pVLM-F	GGTACCTTCTAGAATTCATGGACTCATCCAGGACAATC
pVLM-R	ATACAATCCTTTCAGGAAAGGAAGCGGAGCTACTAACCAGCCTGCTGAAGCAGGCTGGA
pVLNP-F	CCTGCTGAAGCAGGCTGGAGACGTGGAGGAGAACCCTGACCTTCGTCTGTTTTCGACGAATAC
pVLNP-R	GACACTGACTGGGGGTACTGAGCGGCCGCTGCAGATC

Note: The underscored regions of the primer indicate the homology required for recombination.

**Table 2:** Primers used for qRT-PCR.

Primer Name	Primer Sequence (5′-3′)
IFN-γ-F	AAGCGTCATTGAATCACACC
IFN-γ-R	CGAATCAGCAGCGACTCCTT
IL-2-F	TTCAATTGGAAGATGCTGAGA
IL-2-R	ATCATCGAATTGGCACTCAA
IL-4-F	TTTTGAACGAGGTCACAGGA
IL-4-R	AGCCCTACAGACGAGCTCAC
β-actin-F	CATCCGTAAAGACCTCTATGCCAAC
β-actin-R	ATGGAGCCACCGATCCACA

## Data Availability

The data presented in this study are available on request from the corresponding author.
